# Prior contact lens wear reduces susceptibility of the superficially-injured cornea to bacterial adhesion

**DOI:** 10.1371/journal.pone.0332009

**Published:** 2026-05-27

**Authors:** Yujia Yang, Orneika Flandrin, Sara J. Abboud, Eric Jedel, David J. Evans, Suzanne M. J. Fleiszig

**Affiliations:** 1 Herbert Wertheim School of Optometry & Vision Science, University of California, Berkeley, California, United States of America; 2 Graduate Program in Infectious Diseases and Immunity, University of California, Berkeley, California, United States of America; 3 College of Pharmacy, Touro University California, Vallejo, California, United States of America; 4 Graduate Groups in Vision Science, Microbiology, and Infectious Diseases & Immunity, University of California, Berkeley, California, United States of America; Harvard Medical School, UNITED STATES OF AMERICA

## Abstract

Contact lens wear in humans and mice is consistently associated with asymptomatic corneal parainflammation. Here, we tested the hypothesis that the corneal response to lens wear alone can function to protect it against bacterial adhesion enabled by superficial injury. One eye only of mT/mG-LysMcre mice (cell membranes red; Lyz2^+^ cells green) wore a contact lens for 4–6 days. After lens removal, mice were anesthetized and both corneas superficially-injured before bacterial inoculation with either a mouse eyelid commensal (*Macrococcus epidermidis*) or a corneal pathogen (*Pseudomonas aeruginosa*). Inoculation was repeated hourly for 4 hours under anesthesia before euthanasia. Enucleated eyes were fixed overnight, and adherent bacteria visualized using a universal 16S rRNA-targeted FISH probe (*M. epidermidis*) or Blue Fluorescent Protein (*P. aeruginosa*). Confocal imaging and Imaris software were used to quantify bacterial adhesion and location in the epithelium, and also the number, location and morphology of Lyz2^+^ cells. For both commensal and *P. aeruginosa*, prior lens wear resulted in reduced adhesion to the superficially-injured corneas (~46% and ~57% respectively). In both instances this correlated with increased numbers of corneal Lyz2^+^ cells. Other details differed for the two bacterial types. For the commensal, prior lens wear resulted in bacteria penetrating deeper into the epithelium versus contralateral eyes, with Lyz2^+^ cells extending their processes further into the epithelium and localizing closer to the cornea surface. For *P. aeruginosa*, prior lens wear resulted in adherent bacteria closer to the cornea surface, and Lyz2^+^ cells moving further away from it. Moreover, while overall Lyz2^+^ cell sphericity increased for *P. aeruginosa* with prior lens wear versus contralateral eyes, it showed no overall change for the commensal. Lyz2^+^ cell volume in the central cornea was decreased for *P. aeruginosa* with prior lens wear but increased for the commensal. Thus, corneal responses to prior lens wear can quantitively reduce bacterial adhesion to superficially-injured corneas correlating with a Lyz2^+^ cell response for both a commensal and a pathogen, with differences in details for the two bacterial types. How continued lens wear supersedes this protective response to promote *P. aeruginosa* infection pathogenesis remains to be determined, as does the relationship to the Lyz2^+^ cell response.

## Introduction

Contact lenses are widely used for vision correction and therapeutic management of numerous eye pathologies [1,2], yet their wear can lead to adverse events, the most serious being sight-threatening corneal infection [3,4]. It is important to understand the mechanisms by which contact lens wear can predispose to infection and other adverse events. In addition to human studies, researchers have developed various lens wearing animal models to study the impact of lens wear on the cornea and ocular surface and to develop lenses with therapeutic value [5–10].

More recently our laboratory developed a murine model of lens wear using lenses custom-designed to fit the mouse eye [11]. Using this model, we showed that mice wearing lenses inoculated with *Pseudomonas aeruginosa*, a leading cause of contact lens-related corneal infection [12,13], developed infectious pathology from 1–13 days after lens insertion [11]. Interestingly, in the absence of bacterial inoculation, lens wear was associated with subclinical inflammatory responses involving increased numbers of CD11c^+^ cells (after 24 h), Lyz2^+^ cells (at 7 days) and Ly6G^+^ cells (5–6 days) [11]. Subsequent studies showed that 24 hours of lens wear also involved increased numbers of MHC-II^+^ cells and γδ T cells [14] and that 6 day Ly6G^+^ cell responses required γδ T cells and IL-17A [15]. Subclinical corneal inflammation induced by 24 hours of lens wear was shown to only resolve at 7 days after lens removal [16]. These lens-induced immune cell changes in mice resembled changes in presumed Langerhans cell density shown in human corneas as early as 2 hours after contact lens insertion [17] and also after 7 days of daily disposable lens wear [18]. We termed the subclinical lens-induced corneal inflammation in mice as parainflammatory as postulated by Dr. Nathan Efron for human wear [19]. However, it is not yet known if these subclinical corneal responses in mice (or in humans) are beneficial or detrimental to corneal health or if they represent a precursor to clinically evident inflammation or infection.

The purpose of the present study was to use the murine model of lens wear to test the hypothesis that corneal responses to lens wear after 4–6 days would have a protective effect against bacterial adhesion, an important first step in the development of infection. Since the murine cornea is normally remarkably resistant to bacterial adhesion [20,21], we utilized a superficial injury (tissue paper blotting) model to promote bacterial adhesion without subsequent epithelium traversal [22], then compared the impact of prior lens wear versus no lens wear on corneal defense against bacterial adhesion. A murine ocular commensal bacterium (*Macrococcus epidermidis*) and the opportunistic pathogen *P. aeruginosa* were tested and differences in bacterial adhesion were correlated with quantitative and morphological changes to corneal Lyz2^+^ immune cells.

## Materials and methods

### Ethics statement

All procedures involving mice were carried out in accordance with a protocol (AUP-2019-06-12322) approved by the Animal Care and Use Committee, University of California, Berkeley which is an AAALAC (Association for Assessment and Accreditation of Laboratory Animal Care) accredited institution. The protocol adheres to PHS policy on the humane care and use of laboratory animals, and the guide for the care and use of laboratory animals. Procedures adhered to the ARVO (Association for Research in Vision and Ophthalmology) statement for the use of animals in ophthalmic vision research.

### Mice

Six-week-old male and female mT/mG-LysMcre mice were used. These mice were derived from the cross (F1) of a mT/mG mouse (all cell membranes, red) with a LysMcre mouse (Lyz2^+^ cells, myeloid-derived, green) allowing visualization of Lyz2^+^ cells (green) within the cornea (red) [11]. A total of 10 mice (20 eyes) were used for the study: 6 mice (12 eyes) for experiments with commensal bacteria, 4 mice (8 eyes) for experiments with *P. aeruginosa* (see below).

### Contact lens fitting

Mice were lightly anesthetized with 1–3% isoflurane using a precision vaporizer (VetEquip Inc., Pleasanton, CA, USA). A custom-made silicone-hydrogel mouse contact lens was fitted onto the right eye of each mouse as previously described [11] while contralateral eyes did not wear a lens. Briefly, after anesthesia, an Elizabethan collar (Kent Scientific, Torrington, CT, USA) was applied to each mouse and contact lenses were fitted using a Handi-Vac suction pen (Edmund Optics, Barrington, NJ, USA). After lens insertion, mice were individually housed with Pure-o'Cel paper bedding (The Andersons Inc., Maumee, OH, USA), and lenses worn for 4–6 days. Mice were checked daily for well-being and continued lens wear.

### Bacterial adhesion assays

Two bacterial species were used for bacterial adhesion assays. A coagulase-negative murine eyelid commensal (*Macrococcus epidermidis*) and an invasive strain of the opportunistic pathogen *Pseudomonas aeruginosa* expressing Blue Fluorescent Protein (PAO1F-EBFP2) [23] for imaging purposes. Bacteria were prepared by growth on a Tryptic Soy Agar (TSA) plate for ~16 hours at 37°C then suspended in PBS to a concentration of ~10^11^ colony-forming units (CFU)/ml. Following contact lens wear, mice were anesthetized by intraperitoneal injection of ketamine (80–100 mg/Kg) and dexmedetomidine (0.25–0.5 mg/Kg). For each mouse both corneas were superficially-injured (blotted) using a Kimwipe^TM^ tissue paper to promote bacterial adhesion [22] then both inoculated with 5 μl of bacterial suspension once every hour for 4 hours to allow a comparison of bacterial adhesion to the prior lens wear cornea versus the no lens wear cornea. Mice remained anesthetized for the entire 4 hour incubation period and were kept warm on a heated pad. At the end of the incubation period, mice were immediately euthanized by intraperitoneal injection of ketamine (80–100 mg/Kg) and xylazine (5–10 mg/Kg) followed by cervical dislocation. Mice did not wake before euthanasia and no animals died during the 4 hour incubation. After euthanasia, eyeballs were enucleated, rinsed with PBS, fixed in 2% paraformaldehyde (PFA) overnight at 4°C then processed for quantitative confocal imaging as described below.

### Fluorescence in-situ hybridization (FISH) labeling of commensal bacteria

FISH was used to visualize adherent commensal bacteria on whole eyeballs following fixation as previously described [21]. Briefly, eyeballs were washed in PBS, 80% ethanol, and 95% ethanol sequentially for 10 minutes each at room temperature (RT) followed by incubation in hybridization buffer (0.9 M NaCl, 20 mM Tris-HCl, and 0.01% SDS) at 55°C for 30 minutes. Then a universal 16S rRNA-targeted gene probe [Alexa647]-GCTGCCTCCCGTAGGAGT-[Alexa647] (Eurofins Genomics) was added to the eyeballs at a final concentration of 100 nM before overnight incubation at 55°C. Eyeballs were washed in washing buffer (0.9 M NaCl, 20 mM Tris-HCl) for three times with 10 minutes each at RT and stored in PBS before imaging.

### Imaging and processing

Whole eyeballs were imaged using an Olympus FluoView confocal microscope with a 488 nm laser used for detection of Lyz2^+^-GFP cells and 559 nm laser for the detection of red cell membranes. *P. aeruginosa* expressing BFP was detected using a 405 nm laser. FISH-labeled bacteria were detected using a 635 nm laser. Z stack images were acquired at a 0.8 μm step-size and an aspect ratio of 1024 μm x 1024 μm for bacteria, or at a 1.5 μm step-size and 800 μm x 800 μm aspect ratio for immune cells. Acquired Z stacks were reconstructed as 3-D images using Imaris software (Oxford Instruments, Bitplane AG, Zurich, Switzerland). For adherent bacteria, the ‘Spots’ function was used for quantification and bacterial penetration of the epithelium was calculated with reference to the corneal epithelium surface. Due to the expected wider distribution of Lyz2^+^ cells in the cornea, the ‘Surface’ function was first used to render the corneal endothelium layer and Lyz2^+^ cell location calculated with reference to the endothelium. Lyz2^+^ cell volume and sphericity were also measured.

### Statistical analysis

Each group contained 4–6 eyes. For each eye, one center and two peripheral fields of view were imaged. Quantified bacterial adhesion or immune cell responses in the central cornea were shown in the figures by one data point and for the peripheral cornea, one data point averaged from two peripheral fields of view was shown. A normality test was performed on the accumulated quantitative data which were presented as either the mean ± standard error of the mean (SEM) for parametric data or the median with upper and lower quartiles (median [Q1-Q3]) for non-parametric data as indicated in each figure. For parametric data, a Student’s t-test was applied and for non-parametric data, either a Mann-Whitney U test or Wilcoxon signed-rank test were applied. P < 0.05 was considered significant. Prism 10 was used (GraphPad Software, Boston, MA, USA).

## Results

### Prior contact lens wear reduces commensal bacteria adhesion to the cornea but increases bacterial depth of penetration

**Fig 1:**
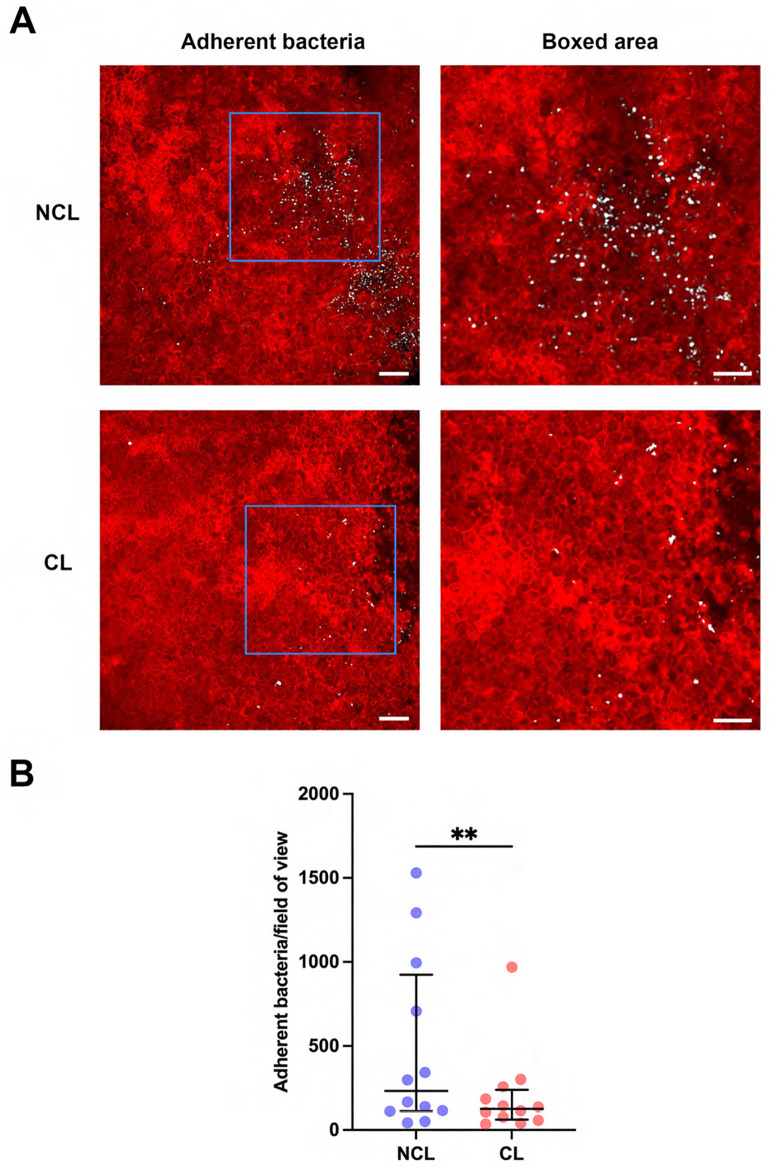
Prior contact lens wear is associated with reduced adherence of commensal bacteria to a superficially-injured mouse cornea. A) Images showing superficially-injured corneas of mT/mG-LysMcre mice (red cell membranes) with (CL) or without (NCL) prior contact lens wear (4-6 days). Adherent bacteria, *Macrococcus epidermidis* (white), were detected using a 16S rRNA-targeted FISH probe. Right panels show higher magnification of the blue boxed areas with adherent bacteria. Scale bars: left 50 µm, right 30 µm. B) Quantification of adherent bacteria per field of view, showing a significant reduction of the median bacterial adhesion with prior lens wear. **P < 0.01 (Wilcoxon signed-rank test).

**Fig 2:**
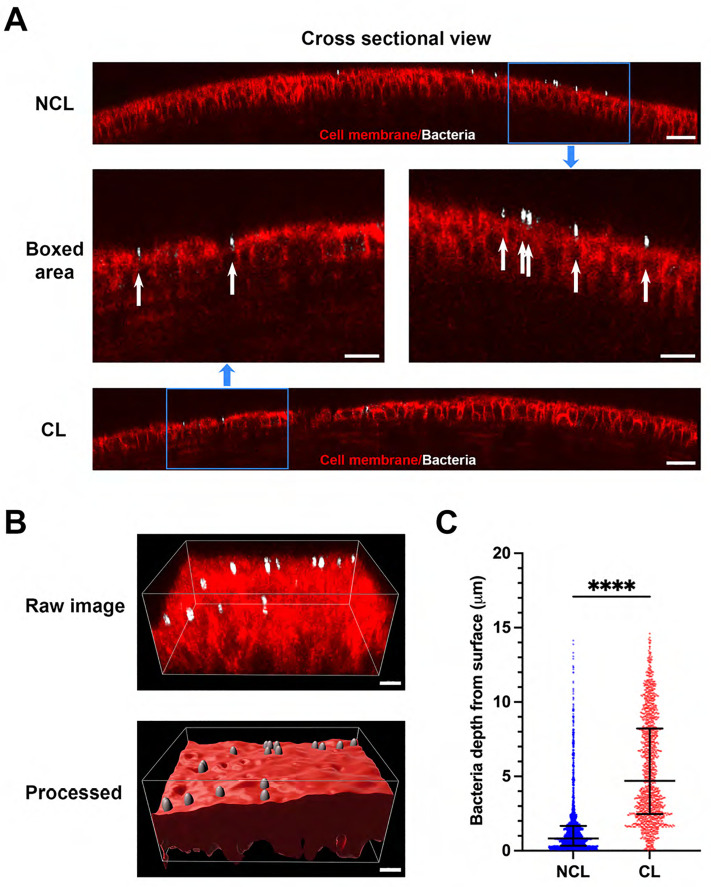
Prior contact lens wear is associated with deeper entry of the corneal epithelium by commensal bacteria adhering after superficial injury. A) Spatial positions of adherent bacteria (white) on the superficially-injured corneal epithelium (red) shown in cross-sectional view with (CL) and without (NCL) prior lens wear. Middle panels showed blue boxed areas from upper and lower panels in a higher magnification with bacteria indicated by white arrows. Scale bars: upper and lower 30 µm, middle 20 µm. B) Imaris ‘Spots’ rendering of adherent bacteria and surface rendering of the corneal epithelium with depth of penetration measured by distance of the spots to the surface. Scale bar 10 µm. C) Quantification of median bacterial depth from the epithelium surface showing deeper penetration with prior contact lens wear. ****P < 0.0001 (Mann-Whitney U test).

We first explored if prior lens wear would impact adhesion of the bacterial commensal to the superficially-injured cornea. Fig 1 shows significantly fewer bacteria (white) adhered to corneas (red) that had worn a contact lens versus contralateral eyes, a ~ 46% decrease (P = 0.009, Wilcoxon signed-rank test). Despite fewer adherent bacteria, however, prior lens-wearing corneas showed greater depth of bacterial entry into the epithelium. Fig 2 shows the increased distance of adherent bacteria from the epithelium surface with prior lens wear: NCL 0.83 [0.34–1.67] vs. CL 4.71 [2.47–8.22] μm (P < 0.0001, Mann-Whitney U test).

### Prior contact lens wear induced corneal Lyz2 + cell recruitment with cells moving closer to the epithelium surface

**Fig 3:**
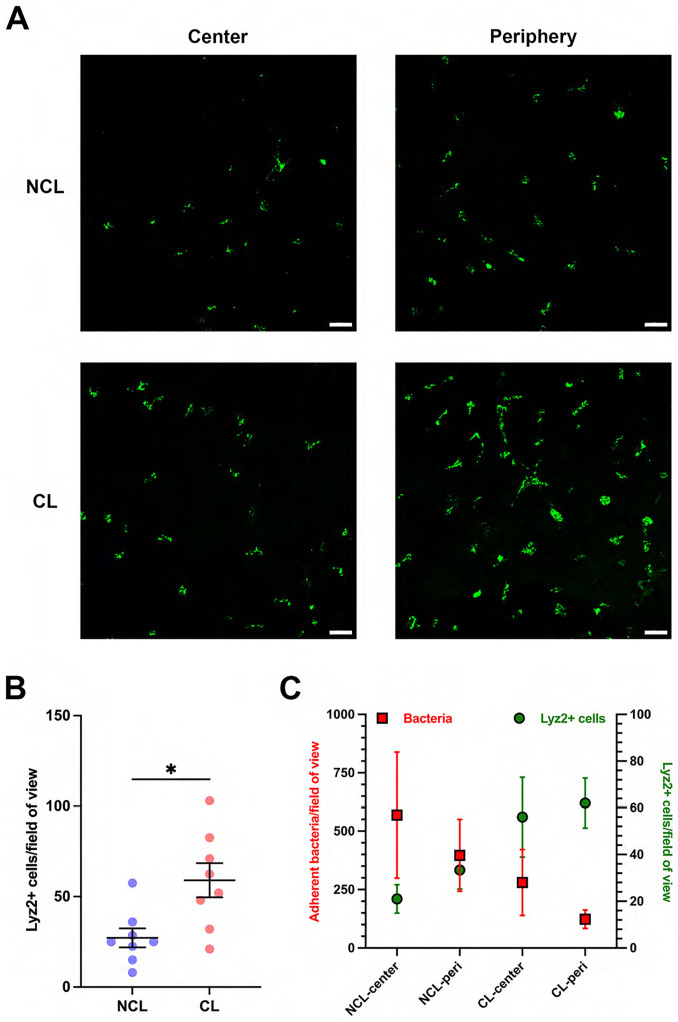
Prior contact lens wear is associated with greater Lyz2^+^ cell numbers in superficially-injured corneas after inoculation with commensal bacteria versus no lens wear. A) Maximum intensity z-projection of the GFP signal from Lyz2^+^ cells in the mT/mG-LysMcre mouse cornea after superficial-injury and commensal bacteria inoculation (4 hours). Lyz2^+^ cell distributions are shown in central and peripheral areas of the cornea with (CL) or without (NCL) lens wear (4−6 days). Scale bar 50 µm. B) Corneal Lyz2^+^ cell numbers per field of view with or without lens wear. *P < 0.05 (Student’s t-test). C) Correlation analysis shows a strong negative correlation between commensal bacteria adhesion and Lyz2^+^ cell numbers comparing central and peripheral corneas with and without prior lens wear (Pearson correlation coefficient r = − 0.967, P = 0.033).

Since we previously showed Lyz2^+^ cell infiltration in lens wearing corneas after 6 or 7 days wear in the absence of deliberate bacterial inoculation or superficial injury [11], we compared Lyz2^+^ cell numbers and morphology in prior lens wear versus no lens wear corneas after superficial injury and inoculation with commensal bacteria. A ~ 54% increase in corneal Lyz2^+^ cells was observed in prior lens-wearing corneas vs. no lens wear contralateral eyes (Fig 3A, B) (P = 0.011, Student’s t-test). More Lyz2^+^ cells were found in the peripheral vs. central cornea in both groups (Fig 3A, B). Increased Lyz2^+^ cell numbers closely correlated with decreased adherence of commensal bacteria when analyzing central and peripheral corneas in both groups (Fig 3C) (Pearson correlation coefficient r = − 0.967, P = 0.033).

**Fig 4:**
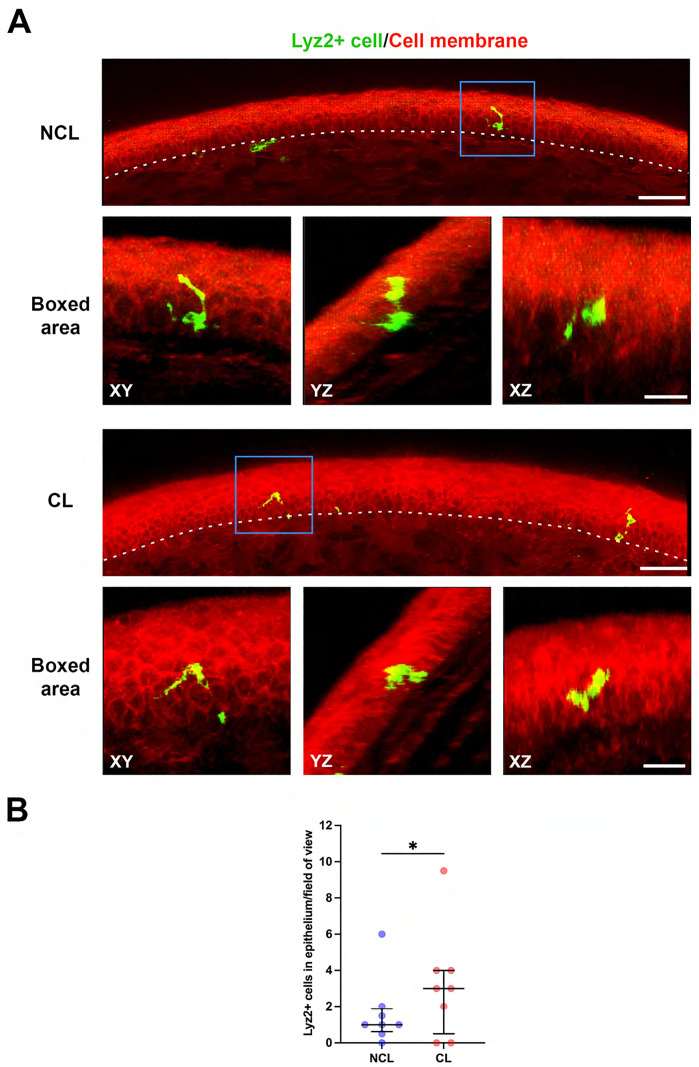
Prior lens wear is associated with more Lyz2^+^ cell migration into the corneal epithelium. A) Extended sectional view (15 µm) of a superficially-injured mT/mG-LysMcre mouse peripheral cornea after commensal bacteria inoculation (4 hours) showing Lyz2^+^ cells (green) inside or extended processes into the corneal epithelium (basal membrane indicated by white dotted lines) with (CL) and without (NCL) prior lens wear. Representative three-dimensional images of Lyz2^+^ cells in the corneal epithelium indicated by blue boxes and displayed in XY, YZ and XZ planes at higher magnification. Scale bar 50 µm or boxed area 20 µm. B) Quantification of Lyz2^+^ cell number per field of view showing increased Lyz2^+^ cells inside or extended processes into corneal epithelium with prior contact lens wear. *P < 0.05 (Wilcoxon signed-rank test).

**Fig 5:**
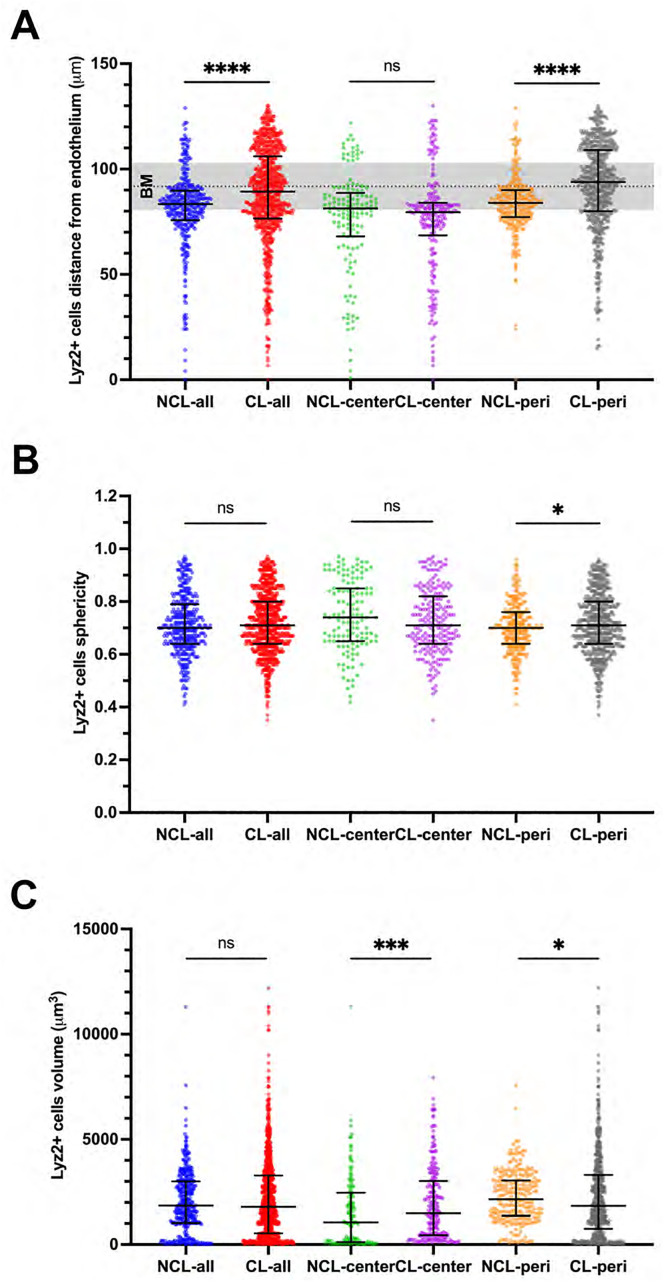
Distributional and morphological changes of Lyz2^+^ immune cells in the superficially-injured cornea after commensal bacteria inoculation. A) Lyz2^+^ cells showed increased migration towards the corneal epithelium (i.e., further away from the endothelium [Y axis = 0]) with migration through the epithelium basal membrane (BM) in prior lens wear corneas, specifically in the peripheral (peri) cornea. B) Lyz2^+^ cells showed little difference in sphericity with and without prior lens wear albeit with a statistically significant increase in the peripheral cornea with prior lens wear (1 = most spherical). C) Lyz2^+^ cell volume showed opposing changes with prior lens wear when comparing the central (increased) vs. peripheral (decreased) cornea versus no lens wear. *P < 0.05, ***P < 0.001, ****P < 0.0001, ns = not significant (Mann-Whitney U test).

Examination of Lyz2^+^ cell location in superficially-injured corneas after commensal bacteria inoculation showed that some of these cells were either within the epithelium or extending processes into the epithelium in both prior lens wear and no lens wear groups (Fig 4A). These epithelium-associated Lyz2^+^ cells were more frequently observed in prior lens-wearing corneas (Fig 4B) (P = 0.031, Wilcoxon signed-rank test). Lyz2^+^ cells were also closer to the corneal epithelium surface, thus further away from the endothelium, in prior lens-wearing corneas versus no lens wear with these differences apparent only in the peripheral cornea (Fig 5A). For example, Lyz2^+^ cell distance from the corneal endothelium (Y axis = 0) in the peripheral cornea was: NCL 83.9 [77.2–90.0] vs. CL 93.8 [80.0–109.0] µm (P < 0.0001, Mann-Whitney U test). Analysis of Lyz2^+^ cell morphology showed a small increase in cell sphericity in prior lens-wear corneas versus no lens wear in peripheral cornea cells (Fig 5B). For example, in the peripheral cornea with 1.0 being most spherical: NCL 0.70 [0.64–0.76] versus CL 0.71 [0.64–0.80] (P = 0.04, Mann-Whitney U test). However, overall changes in cell sphericity were not significant (Fig 5B). No overall change in Lyz2^+^ cell volume was detected between prior lens-wear and no lens wear corneas after commensal inoculation (Fig 5C), although opposing differences were observed if central and peripheral corneas were compared. For the central cornea, median cell volume increased with prior lens wear: NCL 1,053 [112–2,470] vs. CL 1,493 [440–3,026] µm^3^ (P = 0.001, Mann-Whitney U test). In contrast, peripheral cornea median cell volume decreased with prior lens wear: NCL 2,152 [1,357–3,046] vs. CL 1,844 [744 − 3,309] µm^3^ (P = 0.034, Mann-Whitney U test, Fig 5C).

### Prior lens wear reduces *P. aeruginosa* adhesion to the cornea and its epithelium penetration

**Fig 6:**
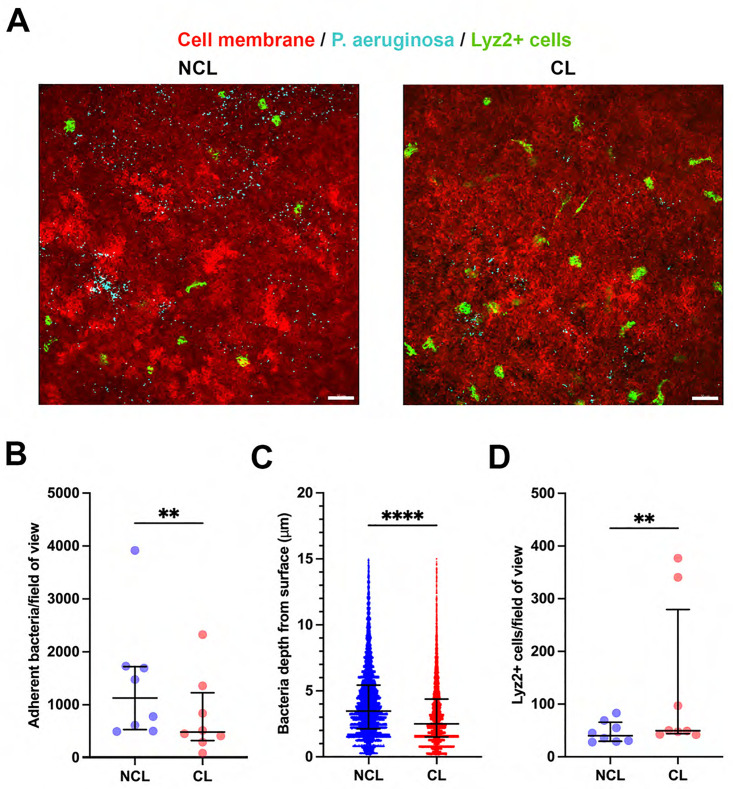
Prior contact lens wear reduces *P. aeruginosa* adhesion and epithelium penetration. A) Representative images show fewer adherent *P. aeruginosa* (Cyan) on the cornea of a mT/mG- LysMcre mouse after superficial-injury and bacterial inoculation (4 hours) with prior lens wear (4−6 days) vs. no lens wear. Scale bar 50 µm. B) Quantification showing significantly reduced adherent *P. aeruginosa* per field of view with prior lens wear. C) Reduced penetration of *P. aeruginosa* into the corneal epithelium with prior lens wear, and D) Increased Lyz2^+^ cells per field of view with prior lens wear. For B and D, **P < 0.01 (Wilcoxon signed-rank test). For C, ****P < 0.0001 (Mann-Whitney U test). E) Correlation analysis shows a negative correlation between *P. aeruginosa* adhesion and Lyz2^+^ cell numbers comparing central and peripheral corneas with and without prior lens wear, but the P value was not significant (Pearson correlation coefficient r = − 0.774, P = 0.226).

We next tested the effect of prior lens wear on susceptibility of the superficially-injured cornea to the opportunistic pathogen *Pseudomonas aeruginosa*. As observed for commensal bacteria, fewer *P. aeruginosa* adhered to the cornea with prior contact lens wear vs. no lens wear, a ~ 57% decrease (P = 0.008, Wilcoxon signed-rank test) (Fig 6A, B). In contrast to the commensal, *P. aeruginosa* showed reduced epithelium penetration with prior lens wear, NCL 3.47 [2.13–5.43] vs. CL 2.50 [1.50–4.38] µm (P < 0.0001, Mann-Whitney U test) (Fig 6C). Reduced *P. aeruginosa* adhesion with prior lens wear was also associated with increased Lyz2^+^ cell numbers in those corneas (~1.2-fold) (P = 0.008, Wilcoxon signed-rank test) (Fig 6D). However, in contrast to the commensal, the correlation analysis between reduced bacterial adhesion and increased Lyz2 + cell numbers for central and peripheral corneas for both groups was not significant (Fig 6E) (Pearson correlation coefficient r = − 0.774, P = 0.226).

**Fig 7:**
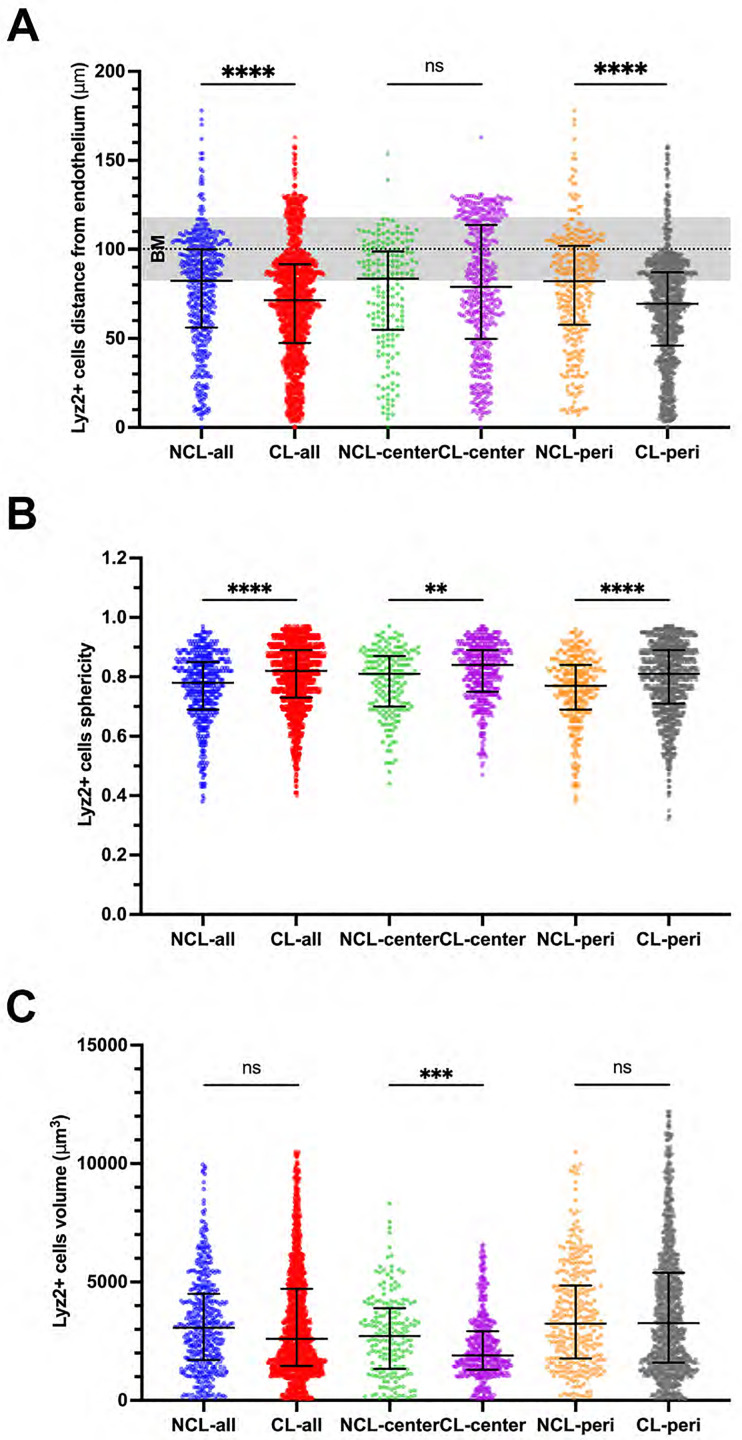
Distributional and morphological changes of Lyz2^+^ immune cells in a superficially-injured cornea after *P. aeruginosa* inoculation. A) Lyz2^+^ cells were further away from the corneal epithelium surface (i.e., closer to the corneal endothelium [Y axis = 0]) with prior lens wear corneas but only in the peripheral cornea. BM = Basement Membrane of the epithelium. B) Lyz2^+^ cells were more spherical in prior lens wear corneas, and C) Lyz2 + cells showed a decrease in cell volume with prior lens wear. *P < 0.05, ***P < 0.001, ****P < 0.0001, ns = not significant (Mann-Whitney U test).

Fig 7 shows Lyz2^+^ cell distribution and morphology changes in the superficially-injured cornea after *P. aeruginosa* inoculation with and without prior lens wear. Most Lyz2^+^ cells were further away from the epithelium surface, thus closer to the endothelium, with prior lens wear: Overall NCL 82.4 [56.2–100.0] vs. CL 71.4 [47.5–91.7] µm from the endothelium (Y axis = 0) (Fig 7A) (P < 0.0001, Mann-Whitney U test). That difference reflected peripheral cornea Lyz2^+^ cells. Although a sub-group in the central cornea appeared to migrate towards the epithelium with prior lens wear (see Fig 7A), that difference not significant vs. no lens wear. Lyz2^+^ cells were more spherical in prior lens wear corneas versus no lens wear, overall and in both the central and peripheral cornea: for example in the central cornea (NCL 0.81 [0.70–0.87] vs. CL 0.84 [0.75–0.89]) P < 0.001 (Mann-Whitney U test) (Fig 7B). Lyz2^+^ cells were smaller in volume with prior lens wear but only in the central cornea (NCL 2,722 [1,331−3,891] vs. CL 1,896 [1,297−2,922] µm^3^ (P = 0.001, Mann-Whitney U test) (Fig 7C).

## Discussion

Here, we used a murine model of contact lens wear to study the impact of 4–6 days prior lens wear on corneal defenses against bacterial adhesion using both commensal and pathogenic bacteria. To enable bacterial adhesion to the otherwise resistant corneal surface, tissue paper blotting was used [22]. The results showed that prior lens wear enabled a significant protective effect against bacterial adhesion of both the commensal and pathogenic bacteria, in both cases associated with increased Lyz2^+^ cell numbers. Thus, prior lens wear over 4–6 days has a protective effect against adhesion of two very different bacteria: a murine commensal (Gram-positive) and a versatile opportunistic pathogen (Gram-negative) and therefore the modification of one or more corneal defenses against bacterial adhesion over the prior lens wearing period.

While prior lens wear protected against bacterial adhesion for both types of bacteria, differences in the details were noted. For example, the adherent bacteria penetrated further into the epithelium for the commensal, while *P. aeruginosa* instead showed a reduced ability to penetrate. For the commensal, the Lyz2^+^ cells moved closer to the adherent bacteria and in the central cornea were larger compared to when a lens had not been worn, whereas for *P. aeruginosa* the Lyz2^+^ cells were further away and smaller in the central cornea. Lyz2^+^ cells were more spherical with prior lens wear versus no lens wear for *P. aeruginosa*, while no overall sphericity changes were observed between groups for the commensal. The differences in bacterial location and in the Lyz2^+^ cell responses (location and morphology) for the commensal versus *P. aeruginosa* challenge suggest different mechanisms might be at play depending on bacterial type.

Lyz2^+^ cells include monocytes, macrophages, a subset of CD11c^+^ (dendritic cells), and neutrophils, each of which can, or has the potential to, modulate the resistance of the cornea to bacterial adhesion. While the role of neutrophils, monocytes and macrophages in responding to and defending already infected corneas against pathogens such as *P. aeruginosa* is well established [24–27], it is unclear whether the altered Lyz2^+^ responses to superficial injury/bacteria induced by prior lens wear are actually involved in protection against adhesion, or whether they are simply correlative? Answers to this important question would be facilitated by further studies of Lyz2^+^ cell responses in this model that identify specific Lyz2^+^ cell type(s) present, along with their state of activation and/or cellular stress that could be inferred by the observed morphology changes.

Directly relevant to this study, we have also used the same superficial injury/*P. aeruginosa* inoculation model without prior lens wear to study CD11c^+^ cell responses and found them migrating *towards* the *P. aeruginosa* adhering to the corneal surface. Depleting the CD11c^+^ cells further enhanced bacterial adhesion [28] showing that CD11c^+^ cells can protect superficially-injured corneas against *P. aeruginosa* adhesion. Here, we introduced prior lens wear to that superficial injury/*P. aeruginosa* challenge model and found that the Lyz2^+^ cells (a subset of which are CD11c^+^ cells) were now *further away* from the adherent bacteria compared to no prior lens wear despite prior lens wear protecting against *P. aeruginosa* adhesion. This suggests that the role of immune cells in protecting superficially-injured corneas against *P. aeruginosa* is likely to be complex and might even depend upon circumstances. Previously, we showed that Ly6G^+^ cells (likely neutrophils) infiltrate the cornea 5–6 days into uninoculated lens wear in this murine contact lens model [11]. Thus, neutrophils (also Lyz2^+^) are another potential candidate for involvement in countering bacterial adhesion after prior lens wear. Other immune cells (e.g., γδ T cells) involved in corneal parainflammatory responses to uninoculated lens wear [11,14–16] may also contribute to the defenses against bacterial adhesion after lens removal shown here. At present, however, a direct connection to lens-induced corneal parainflammation remains to be shown.

It is also possible that factors beyond Lyz2^+^ cell changes are responsible for reduced bacterial adhesion associated with prior lens wear. Various non-immune cell factors have been shown by us, and others, to counter bacterial adhesion at the corneal surface. They include corneal mucins, lectins, antimicrobial peptides (including defensins, keratin derived antimicrobial peptides), DMBT1, surfactant proteins, epithelial cell polarity, and junction-related factors [29–36]. Moreover, we recently reported that corneal defenses against adhesion include their sensory nerves, with TRPA1 involved in countering *P. aeruginosa* [37], and TRPV1 countering both environmental bacteria and *Staphylococcus aureus* [37,38]. Moreover, we have shown that these receptors are also required for contact lens-induced corneal parainflammation and modulation of resident immune cells in the murine lens wear model [14]. This raises the possibility that sensory nerve-associated TRP receptors are also involved in how prior lens wear reduces the propensity for bacterial adhesion. Ultimately, the defense mechanisms involved are likely to be multifactorial possibly involving a combination of sensory nerves, immune cells and changes to intrinsic anti-adhesive or directly antimicrobial defenses. Additional studies will be needed to tease apart the mechanism(s) by which prior lens wear reduces bacterial adhesion to the cornea.

How does prior lens wear protecting the cornea against *P. aeruginosa* adhesion align with the fact that contact lens wear predisposes the cornea to *P. aeruginosa* infection? Here, the lens was deliberately removed before the bacteria were inoculated into the eye. This was to allow us to tease apart the contribution of lens wear itself to defenses against adhesion, the hypothesis being that it induces a protective response based on our observation that there is parainflammation and its significance remained unknown. Removing the lens before adding the bacteria is different from keeping the lens in place whilst the bacteria are present for several reasons. Firstly, contact lens-associated corneal infection in animal models requires a lens deliberately inoculated with *P. aeruginosa* to remain in place on the cornea for up to 13 days in the mouse [11] or for 7–10 days in the rat [10]. Without a lens, *P. aeruginosa* and other bacteria are rapidly cleared from the ocular surface [20,21]. Their inability to gain a foothold on the intact healthy adhesion resistant cornea is because “antimicrobial factors” present at the ocular surface/tear fluid can kill them, incapacitate them, aggregate them, repel them and/or manipulate their gene expression [20,21,29,34,35,39–43]. Secondly, we have shown that the posterior surface of lenses in *P. aeruginosa* infected eyes of rats harbored *P. aeruginosa* biofilms, a sign of bacterial adaptation to the ocular surface environment [10]. This adaptation to the ocular surface on a lens (i.e., exposure to both simultaneously) appears to be a key feature in the pathogenesis of contact lens-related infection, as transferring colonized lenses from rat eyes with *P. aeruginosa* infections into eyes of naïve rats enabled infections to develop more quickly (~2 days) [10]. Additionally, we have shown that if *P. aeruginosa* is exposed to tear fluid for several hours (not normally feasible without lens wear due to clearance mechanisms), the bacteria release outer membrane vesicles able to kill epithelial cells at the surface of the cornea, and that this in turn promotes corneal epithelium susceptibility to *P. aeruginosa* adhesion [44]. Also relevant to this discussion, we previously showed that superficial injury to the cornea alone does not enable infection susceptibility despite it enabling bacterial adhesion [22]. This is because there are deeper layer defenses in place that prevent adherent bacteria from progressing further and superficial injury alone is insufficient to compromise them [28]. Thus, there are many factors contributing to the pathogenesis of contact lens-related corneal infection beyond bacterial adhesion, including changes to both the bacteria and host while a lens remains *in situ* [45]. As such, results of this study showing reduced bacterial adhesion to corneas that had previously worn a lens adds a small piece to the complex puzzle of how the cornea adapts to lens wear and responds to microbes, information that will help to eventually understand and prevent pathogenesis of contact lens related and other infections.

In conclusion, contact lens wear in humans causes both asymptomatic parainflammation and acute corneal infection. While animal models of lens wear can reproduce both events, the relationship between them remains unclear. The present study adds to our knowledge by showing that 4–6 days of lens wear in mice, which enables parainflammation, can render the cornea less susceptible to adhesion by either a commensal or pathogenic *P. aeruginosa* if inoculated after the lens is removed. This differs from events occurring if the lens is left in place, which instead leads to infection. Whether and how the increased corneal Lyz2^+^ immune cell numbers and changes to their location and morphology are involved in the protective phenotype for each bacterial type remains to be determined. It would also be of value to determine the impact of a cytotoxic strain of *P. aeruginosa* (versus invasive strain used here) on corneal adhesion levels observed after prior lens wear. Follow-up studies will also be needed to understand how continuing lens wear during *P. aeruginosa* challenge undermines this defensive response to lead to infection.

## References

[1] Wang Y, Jacobs DS. Role of therapeutic contact lenses in management of corneal disease. Curr Opin Ophthalmol. 2022;33(4):306–10. doi: 10.1097/ICU.0000000000000859 35779054

[2] Lim L, Lim EWL. Therapeutic Contact Lenses in the Treatment of Corneal and Ocular Surface Diseases-A Review. Asia Pac J Ophthalmol (Phila). 2020;9(6):524–32. doi: 10.1097/APO.0000000000000331 33181548

[3] Stapleton F, Keay L, Jalbert I, Cole N. The epidemiology of contact lens related infiltrates. Optom Vis Sci. 2007;84(4):257–72. doi: 10.1097/OPX.0b013e3180485d5f 17435509

[4] Green M, Apel A, Stapleton F. A longitudinal study of trends in keratitis in Australia. Cornea. 2008;27(1):33–9. doi: 10.1097/ICO.0b013e318156cb1f 18245964

[5] Zhang Y, Gabriel MM, Mowrey-McKee MF, Barrett RP, McClellan S, Hazlett LD. Rat silicone hydrogel contact lens model: effects of high- versus low-Dk lens wear. Eye Contact Lens. 2008;34(6):306–11. doi: 10.1097/ICL.0b013e3181891421 18997538 PMC4274951

[6] Vijay AK, Sankaridurg P, Zhu H, Willcox MDP. Guinea pig models of acute keratitis responses. Cornea. 2009;28(10):1153–9. doi: 10.1097/ICO.0b013e3181a87a0b 19770709

[7] Dutta D, Vijay AK, Kumar N, Willcox MDP. Melimine-Coated Antimicrobial Contact Lenses Reduce Microbial Keratitis in an Animal Model. Invest Ophthalmol Vis Sci. 2016;57(13):5616–24. doi: 10.1167/iovs.16-19882 27768798

[8] Ladage PM, Yamamoto K, Ren DH, Li L, Jester JV, Petroll WM, et al. Proliferation rate of rabbit corneal epithelium during overnight rigid contact lens wear. Invest Ophthalmol Vis Sci. 2001;42(12):2804–12. 11687521

[9] Wei C, Zhu M, Petroll WM, Robertson DM. Pseudomonas aeruginosa infectious keratitis in a high oxygen transmissible rigid contact lens rabbit model. Invest Ophthalmol Vis Sci. 2014;55(9):5890–9. doi: 10.1167/iovs.14-14235 25125601 PMC4168740

[10] Tam C, Mun JJ, Evans DJ, Fleiszig SMJ. The impact of inoculation parameters on the pathogenesis of contact lens-related infectious keratitis. Invest Ophthalmol Vis Sci. 2010;51(6):3100–6. doi: 10.1167/iovs.09-4593 20130275 PMC2891469

[11] Metruccio MME, Wan SJ, Horneman H, Kroken AR, Sullivan AB, Truong TN, et al. A novel murine model for contact lens wear reveals clandestine IL-1R dependent corneal parainflammation and susceptibility to microbial keratitis upon inoculation with Pseudomonas aeruginosa. Ocul Surf. 2019;17(1):119–33. doi: 10.1016/j.jtos.2018.11.006 30439473 PMC6365008

[12] Hatami H, Ghaffari Jolfayi A, Ebrahimi A, Golmohammadi S, Zangiabadian M, Nasiri MJ. Contact Lens Associated Bacterial Keratitis: Common Organisms, Antibiotic Therapy, and Global Resistance Trends: A Systematic Review. Front Ophthalmol (Lausanne). 2021;1:759271. doi: 10.3389/fopht.2021.759271 38983972 PMC11182260

[13] Stapleton F. Contact lens-related corneal infection in Australia. Clin Exp Optom. 2020;103:408–17. 32363626 10.1111/cxo.13082

[14] Datta A, Lee JH, Flandrin O, Horneman H, Lee J, Metruccio MME, et al. TRPA1 and TPRV1 Ion Channels Are Required for Contact Lens-Induced Corneal Parainflammation and Can Modulate Levels of Resident Corneal Immune Cells. Invest Ophthalmol Vis Sci. 2023;64(11):21. doi: 10.1167/iovs.64.11.21 37585189 PMC10434714

[15] Datta A, Truong T, Lee JH, Horneman H, Flandrin O, Lee J, et al. Contact lens-induced corneal parainflammation involving Ly6G+ cell infiltration requires IL-17A and γδ T cells. Ocul Surf. 2023;28:79–89. doi: 10.1016/j.jtos.2023.02.004 36758675 PMC10406967

[16] Datta A, Lee JH, Truong T, Flandrin O, Yang Y, Evans DJ, et al. Persistence of Contact Lens-Induced Corneal Parainflammation Following Lens Removal. Invest Ophthalmol Vis Sci. 2024;65(3):8. doi: 10.1167/iovs.65.3.8 38466283 PMC10929745

[17] Alzahrani Y, Pritchard N, Efron N. Changes in corneal Langerhans cell density during the first few hours of contact lens wear. Cont Lens Anterior Eye. 2016;39(4):307–10. doi: 10.1016/j.clae.2016.02.008 26923921

[18] Alzahrani Y, Colorado LH, Pritchard N, Efron N. Longitudinal changes in Langerhans cell density of the cornea and conjunctiva in contact lens-induced dry eye. Clin Exp Optom. 2017;100(1):33–40. doi: 10.1111/cxo.12399 27353750

[19] Efron N. Contact lens wear is intrinsically inflammatory. Clin Exp Optom. 2017;100(1):3–19. doi: 10.1111/cxo.12487 27806431

[20] Mun JJ, Tam C, Kowbel D, Hawgood S, Barnett MJ, Evans DJ, et al. Clearance of Pseudomonas aeruginosa from a healthy ocular surface involves surfactant protein D and is compromised by bacterial elastase in a murine null-infection model. Infect Immun. 2009;77(6):2392–8. doi: 10.1128/IAI.00173-09 19349424 PMC2687330

[21] Wan SJ, Sullivan AB, Shieh P, Metruccio MME, Evans DJ, Bertozzi CR, et al. IL-1R and MyD88 Contribute to the Absence of a Bacterial Microbiome on the Healthy Murine Cornea. Front Microbiol. 2018;9:1117. doi: 10.3389/fmicb.2018.01117 29896179 PMC5986933

[22] Alarcon I, Tam C, Mun JJ, LeDue J, Evans DJ, Fleiszig SMJ. Factors impacting corneal epithelial barrier function against Pseudomonas aeruginosa traversal. Invest Ophthalmol Vis Sci. 2011;52(3):1368–77. doi: 10.1167/iovs.10-6125 21051692 PMC3101686

[23] Kumar NG, Nieto V, Kroken AR, Jedel E, Grosser MR, Hallsten ME, et al. Pseudomonas aeruginosa Can Diversify after Host Cell Invasion to Establish Multiple Intracellular Niches. mBio. 2022;13(6):e0274222. doi: 10.1128/mbio.02742-22 36374039 PMC9765609

[24] St Leger AJ, Desai JV, Drummond RA, Kugadas A, Almaghrabi F, Silver P, et al. An Ocular Commensal Protects against Corneal Infection by Driving an Interleukin-17 Response from Mucosal γδ T Cells. Immunity. 2017;47(1):148-158.e5. doi: 10.1016/j.immuni.2017.06.014 28709803 PMC5553552

[25] Sun Y, Karmakar M, Roy S, Ramadan RT, Williams SR, Howell S, et al. TLR4 and TLR5 on corneal macrophages regulate Pseudomonas aeruginosa keratitis by signaling through MyD88-dependent and -independent pathways. J Immunol. 2010;185(7):4272–83. doi: 10.4049/jimmunol.1000874 20826748 PMC3392180

[26] Karmakar M, Sun Y, Hise AG, Rietsch A, Pearlman E. Cutting edge: IL-1β processing during Pseudomonas aeruginosa infection is mediated by neutrophil serine proteases and is independent of NLRC4 and caspase-1. J Immunol. 2012;189(9):4231–5. doi: 10.4049/jimmunol.1201447 23024281 PMC3482477

[27] Hazlett LD. Role of innate and adaptive immunity in the pathogenesis of keratitis. Ocul Immunol Inflamm. 2005;13(2–3):133–8. doi: 10.1080/09273940490912362 16019672

[28] Metruccio MME, Tam C, Evans DJ, Xie AL, Stern ME, Fleiszig SMJ. Contributions of MyD88-dependent receptors and CD11c-positive cells to corneal epithelial barrier function against Pseudomonas aeruginosa. Sci Rep. 2017;7(1):13829. doi: 10.1038/s41598-017-14243-w 29062042 PMC5653778

[29] Ricciuto J, Heimer SR, Gilmore MS, Argüeso P. Cell surface O-glycans limit Staphylococcus aureus adherence to corneal epithelial cells. Infect Immun. 2008;76(11):5215–20. doi: 10.1128/IAI.00708-08 18794288 PMC2573382

[30] Argüeso P, Guzman-Aranguez A, Mantelli F, Cao Z, Ricciuto J, Panjwani N. Association of cell surface mucins with galectin-3 contributes to the ocular surface epithelial barrier. J Biol Chem. 2009;284(34):23037–45. doi: 10.1074/jbc.M109.033332 19556244 PMC2755710

[31] AbuSamra DB, Argüeso P. Lectin-Glycan Interactions in Corneal Infection and Inflammation. Front Immunol. 2018;9:2338. doi: 10.3389/fimmu.2018.02338 30349544 PMC6186829

[32] Gipson IK, Spurr-Michaud S, Tisdale A, Menon BB. Comparison of the transmembrane mucins MUC1 and MUC16 in epithelial barrier function. PLoS One. 2014;9(6):e100393. doi: 10.1371/journal.pone.0100393 24968021 PMC4072602

[33] Fleiszig SM, Evans DJ, Do N, Vallas V, Shin S, Mostov KE. Epithelial cell polarity affects susceptibility to Pseudomonas aeruginosa invasion and cytotoxicity. Infect Immun. 1997;65(7):2861–7. doi: 10.1128/iai.65.7.2861-2867.1997 9199460 PMC175402

[34] Redfern RL, Reins RY, McDermott AM. Toll-like receptor activation modulates antimicrobial peptide expression by ocular surface cells. Exp Eye Res. 2011;92(3):209–20. doi: 10.1016/j.exer.2010.12.005 21195713 PMC3049847

[35] Augustin DK, Heimer SR, Tam C, Li WY, Le Due JM, Evans DJ, et al. Role of defensins in corneal epithelial barrier function against Pseudomonas aeruginosa traversal. Infect Immun. 2011;79(2):595–605. doi: 10.1128/IAI.00854-10 21115716 PMC3028852

[36] Ni M, Evans DJ, Hawgood S, Anders EM, Sack RA, Fleiszig SMJ. Surfactant protein D is present in human tear fluid and the cornea and inhibits epithelial cell invasion by Pseudomonas aeruginosa. Infect Immun. 2005;73(4):2147–56. doi: 10.1128/IAI.73.4.2147-2156.2005 15784557 PMC1087391

[37] Wan SJ, Datta A, Flandrin O, Metruccio MME, Ma S, Nieto V, et al. Nerve-associated transient receptor potential ion channels can contribute to intrinsic resistance to bacterial adhesion in vivo. FASEB J. 2021;35(10):e21899. doi: 10.1096/fj.202100874R 34569661 PMC8486357

[38] Flandrin O, Yang Y, Abboud S, Kumar NG, Datta A, Jedel E, et al. TRPV1 Defends the Healthy Murine Cornea Against Staphylococcus aureus Adhesion Independently of Sensory Nerve Firing. Invest Ophthalmol Vis Sci. 2025;66(9):61. doi: 10.1167/iovs.66.9.61 40699192 PMC12306694

[39] Li J, Metruccio MME, Evans DJ, Fleiszig SMJ. Mucosal fluid glycoprotein DMBT1 suppresses twitching motility and virulence of the opportunistic pathogen Pseudomonas aeruginosa. PLoS Pathog. 2017;13(5):e1006392. doi: 10.1371/journal.ppat.1006392 28489917 PMC5440049

[40] McDermott AM, Redfern RL, Zhang B, Pei Y, Huang L, Proske RJ. Defensin expression by the cornea: multiple signalling pathways mediate IL-1beta stimulation of hBD-2 expression by human corneal epithelial cells. Invest Ophthalmol Vis Sci. 2003;44(5):1859–65. doi: 10.1167/iovs.02-0787 12714616 PMC1497872

[41] Fleiszig SM, Zaidi TS, Ramphal R, Pier GB. Modulation of Pseudomonas aeruginosa adherence to the corneal surface by mucus. Infect Immun. 1994;62(5):1799–804. doi: 10.1128/iai.62.5.1799-1804.1994 8168942 PMC186410

[42] Tam C, Mun JJ, Evans DJ, Fleiszig SMJ. Cytokeratins mediate epithelial innate defense through their antimicrobial properties. J Clin Invest. 2012;122(10):3665–77. doi: 10.1172/JCI64416 23006328 PMC3461926

[43] Tabor LM, Grosser MR, Metruccio MMME, Kumar NG, Wu YT, Nieto V, et al. Human tear fluid modulates the Pseudomonas aeruginosa transcriptome to alter antibiotic susceptibility. Ocul Surf. 2021;22:94–102. doi: 10.1016/j.jtos.2021.07.004 34332149 PMC10139757

[44] Metruccio MME, Evans DJ, Gabriel MM, Kadurugamuwa JL, Fleiszig SMJ. Pseudomonas aeruginosa Outer Membrane Vesicles Triggered by Human Mucosal Fluid and Lysozyme Can Prime Host Tissue Surfaces for Bacterial Adhesion. Front Microbiol. 2016;7:871. doi: 10.3389/fmicb.2016.00871 27375592 PMC4891360

[45] Fleiszig SMJ, Kroken AR, Nieto V, Grosser MR, Wan SJ, Metruccio MME, et al. Contact lens-related corneal infection: Intrinsic resistance and its compromise. Prog Retin Eye Res. 2020;76:100804. doi: 10.1016/j.preteyeres.2019.100804 31756497 PMC7237316

